# Clinically relevant variants detected in Chinese children with global developmental delay/intellectual disability: An exome-wide sequencing study

**DOI:** 10.1016/j.gendis.2024.101389

**Published:** 2024-08-09

**Authors:** Yimeng Qiao, Nan Lv, Tongchuan Li, Ye Cheng, Yunqian Li, Jiqiang Dong, Meimei Han, Yang Gu, Qing Shang, Qinghe Xing

**Affiliations:** a Children's Hospital of Fudan University and Institutes of Biomedical Sciences of Fudan University, Shanghai 201102, China; b Henan Key Laboratory of Child Brain Injury, Department of Pediatrics, The 3rd Affiliated Hospital of Zhengzhou University and Institute of Neuroscience, Zhengzhou, Henan 450052, China; c NHC Key Laboratory of Birth Defects Prevention, Henan Key Laboratory of Population Defects Prevention, Zhengzhou, Henan 450002, China; d Children's Hospital Affiliated to Zhengzhou University, Henan Children's Hospital, Zhengzhou Children’s Hospital, Zhengzhou, Henan 450053, China; e Puyang Maternity and Child Care Centers, Puyang, Henan 457005, China; f Shanghai Center for Women and Children's Health, Shanghai 201102, China

Global developmental delay/intellectual disability (GDD/ID) with a prevalence of 1%–3% represents one of the biggest medical and social challenges in our society.^1^ Genetic factors are the main causes of GDD/ID and early diagnosis is crucial to improving the prognosis of GDD/ID children.^2^ Chromosomal microarray analysis, as a first-tier clinical test,^3^ remains limited because of the insufficient to detect small variations while whole exome sequencing (WES) can detect both single-nucleotide variants (SNVs) and copy-number variants (CNVs), effectively improving the diagnostic yield of GDD/ID.^4^ Various adverse risk factors could contribute to GDD/ID and the effects of a genetic variant can vary depending on the presence or absence of adverse preceding events.^5^ Herein, to evaluate the contribution of the genetic etiology to GDD/ID and to investigate the association between known risk factors and genetic etiology in children with GDD/ID, and further, to explore candidate pathogenic genes of GDD/ID, we conducted WES of a Chinese GDD/ID cohort.

We enrolled 211 children with unexplained GDD/ID (Fig. 1A and Table S1), including 179 cases (84.8%) with GDD (<60 months) and 32 (15.2%) ID (>60 months). Among them, 137 (64.9%) were male and 74 (35.1%) were female (Fig. 1B); the patients were aged 3–156 months (average age, 32.5 ± 26.6 months) and had birth weights of 1390–4600 g (average weight, 3226.2 ± 534.5 g).

**Figure 1:**
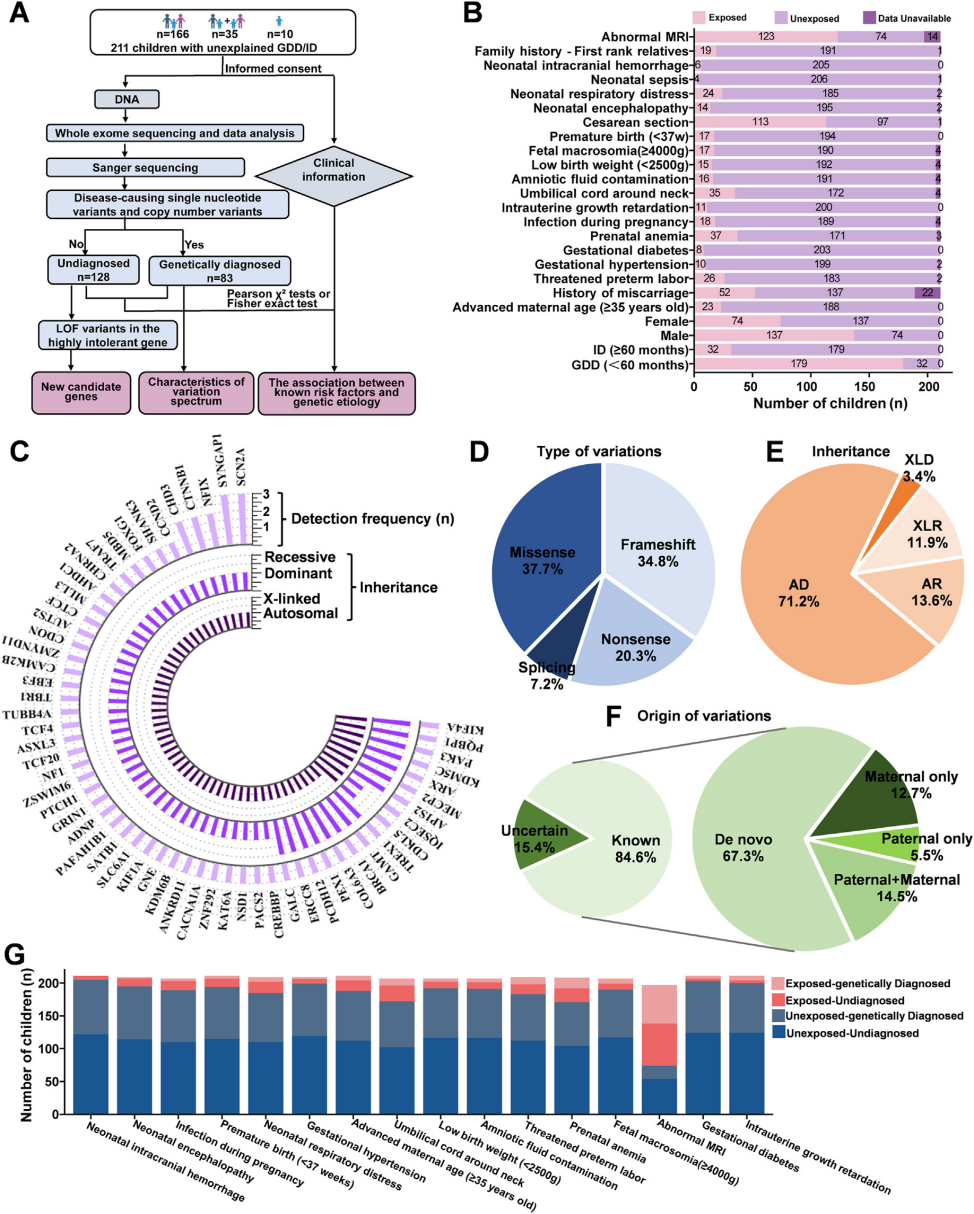
Genetic diagnostic variants in 211 Chinese children with unexplained global developmental delay (GDD)/intellectual disability (ID) by whole exome sequencing. **(A)** The flow chart of exome-wide sequencing study on 211 Chinese children with GDD/ID. **(B)** The clinical characteristics of 211 children with GDD/ID. **(C)** The detection frequency and inheritance of 59 genes. **(D)** Distribution of variation types. **(E)** Distribution of inheritance pattern. **(F)** Distribution of variation origins. The origin of variations in 10 children could not be determined because the samples of their parents were unavailable. **(G)** Genetic diagnostic distribution of GDD/ID children with different risk factors. The detailed data is listed in Table S5.

Finally, 83 of the 211 (39.3%) children with GDD/ID met each of the genetically diagnostic criteria regarding pathogenicity, appropriate inheritance pattern, and disease-phenotype concordance based on a combined analysis of CNVs and SNVs using WES data (Table S2, 3). SNVs alone accounted for 30.8% (65/211) of cases, whereas CNVs alone accounted for 9.0% (19/211). Of the 83 diagnosed cases, 1 carried two pathogenic and likely pathogenic (P/LP) SNVs from different gene loci, and 1 carried both an SNV and a CNV.

By analyzing the SNVs, we established 65 (30.8%) distinct presumptive molecular diagnoses in the 211 patients with GDD/ID (Table S2). In total, we identified 69 P/LP variants across 59 known neurodevelopmental disorder genes (Fig. 1C), including 26 (37.7%) missense, 5 (7.2%) splicing, 14 (20.3%) nonsense, and 24 (34.8%) frameshift variants (Fig. 1D). Forty (58.0%) P/LP variants spanning 36 genes were previously undocumented, including 3 splicing, 8 nonsense, 20 frameshift, and 9 missense variants. In terms of inheritance mode, 42 of 59 genes presented an autosomal dominant (AD) pattern in 48 cases, 8 of 59 genes exhibited an autosomal recessive (AR) pattern in 8 cases, and 9 of 59 genes showed an X-linked (XL) pattern in 9 cases (Fig. 1E). Of these 59 genes, *SCN2A* and *SYNGAP1* were the most mutated genes (*n* = 3), followed by *CHD3*, *CTNNB1* and *NFIX* (2 cases each) (Fig. 1C). Fifty-five genetically diagnosed children were from trios, among which 37 (67.3%) were diagnosed with *de novo* variants, 7 (12.7%) with maternal variants, 3 (5.5%) with parental variants, and 8 (14.5%) with both paternal and maternal variants (Fig. 1F). Of the 48 children diagnosed with AD gene variants, 34 (70.8%) carried *de novo* variants, suggesting that prenatal diagnosis rather than genetic carrier screening is more effective for detecting fetuses with GDD/ID.

As CNVs are important contributors to the etiology of GDD/ID, we further conducted a CNV analysis based on the WES data. Ultimately, 19 P/LP CNVs were detected in 19 children with GDD/ID (Table S3). The pathogenic CNV in 15q11-13 was identified in 4 (1.9%) children as the most frequently detected CNV, indicating that simultaneous analysis of both SNVs and CNVs based on WES data may comprehensively identify the GDD/ID variation landscape.

We detected 2 (0.9%) children with multiple diagnostic variants (Table S2). Case 2633, a 78-month-old male child with severe ID, carried an LP variant in *SATB1* inherited from his mother with ID and an LP variant in *SCN2A* inherited from his father with ID. Case 2590, another 8-month-old male patient with GDD, delayed development of white matter myelin sheath, and widened bilateral frontotemporal subarachnoid space and anterior diaphragmatic cisterna, carried both an LP variant of *ASXL3* and a pathogenic CNV (Table S2, 3). The existence of multiple diagnostic variants can greatly complicate the diagnosis and treatment of GDD/ID. Additionally, among the 83 children who had a genetic diagnosis, 7 (separately carried P/LP variants in *SCN2A*, *GALC*, *ERCC8*, *TREX1* and *GAMT*) could be controlled using medication at an early stage of the disease if they underwent genetic testing (Table S4).

GDD/ID children with the same risk factor exposure may have a similar pathogenesis. Therefore, we examined the differences between the genetic diagnosis rates of children with GDD/ID and various risk factors (Fig. 1G and Table S5). The genetic diagnosis rate of GDD/ID children with neonatal encephalopathy (14.3%) was significantly lower than that of children absent from neonatal encephalopathy (41.5%) (*P* = 0.044; OR = 0.235; 95% CI: 0.051–1.077), indicating that genetic etiology is not the leading cause of neonatal encephalopathy. However, the genetic diagnosis rate of GDD/ID children with abnormal magnetic resonance imaging (MRI) findings (48.0%) was significantly higher than that of unexposed children (27.0 %) (*P*= 0.004; OR = 2.489; 95% CI: 1.335–4.642), suggesting that GDD/ID children with genetic causes are more likely to present with abnormal MRI, which are a secondary response to genetic defects. Moreover, there was no significant difference in the genetic diagnostic rates of subgroups with other risk factors, partially because of the small sample size.

To investigate the potential genetic defects associated with GDD/ID, we further analyzed loss-of-function (LOF) variants in the highly intolerant gene (probability of being LOF intolerant >0.9; upper bound of the oe 90% confidential interval <0.35) in 128 undiagnosed children. We detected 21 LOF variants across 20 highly intolerant genes (Table S6). Of these variants, 2 children carried LOF variants in *SSH2*, and a hemizygous LOF variant in *SMARCA1* was identified. Considering the origin of LOF variants in the highly intolerant gene and ample expression in the brain, *AGAP2* harbored a *de novo* LOF variant, and *SMARCA1* carried a maternally inherited hemizygous variant as two of the top-priority candidate genes associated with GDD/ID.

There are some limitations in this study. First, the small sample size resulted in insufficient guidance for clinical application due to the high genetic heterogeneity of GDD/ID. Second, the existing sample size only has enough statistical power to reveal risk factors associated with molecular diagnostic yield with a large effect size. Third, novel variants detected by WES in our study lack of necessary functional verification. Further functional research on cell models, animal models, and clinical patients will be required to confirm their roles in the etiologies of GDD/ID.

In conclusion, combined analysis of SNVs and CNVs using WES established a genetic diagnosis in 83 of 211 (39.3%) children with GDD/ID. Our results broadened the mutation spectrum of GDD/ID and provided a wealth of available data on genetic studies of GDD/ID. The novelty of our work also lies in the meticulous evaluation of individual risk factors' impact on the diagnostic yield of GDD/ID, which can make up for the deficiency of previous research focusing solely on the collective effects of known risk factors.

## Ethics declaration

This study was approved by the Ethics Committee of the Children's Hospital of Fudan University (2019-070) in accordance with the principles of the Declaration of Helsinki. Statements of informed consent were obtained from the guardians of all children after a full explanation of the procedure. All participants' legal guardians gave informed consent that their clinical details listed in this manuscript could be used for scientific publication.

## Funding

This work was supported by the Shanghai Municipal Commission of Science and Technology Research Project (China) (No. 19JC1411001), the 10.13039/501100012166National Key Research and Development Program from the 10.13039/501100002855Ministry of Science and Technology of the People's Republic of China (No. 2021YFC2700800), the 10.13039/501100001809National Natural Science Foundation of China (No. 31972880, 32170615, 31611130035, 31371274), the National Key Research and Development Plan for Stem Cell and Transformation Research (China) (No. 2017YFA0104202), the Collaborative Innovation Center Project Construction for Shanghai Women and 10.13039/100012422Children's Health, the Open Research Fund of 10.13039/501100018684National Health Commission Key Laboratory of Birth Defects Prevention & Henan Key Laboratory of Population Defects Prevention (China) (No. ZD202309), The Medical Science and Technology Research Program Project of Henan Province, China (No. LHGJ20230368), and the Postdoctoral Research Fund of the Third Affiliated Hospital of 10.13039/501100004605Zhengzhou University (No. BSH20230101).

## CRediT authorship contribution statement

**Yimeng Qiao:** Formal analysis, Funding acquisition, Investigation, Methodology, Writing – original draft, Writing – review & editing. **Nan Lv:** Investigation, Resources, Writing – review & editing. **Tongchuan Li:** Investigation, Resources, Writing – review & editing. **Ye Cheng:** Formal analysis, Investigation, Writing – review & editing. **Yunqian Li:** Investigation, Writing – review & editing. **Jiqiang Dong:** Investigation, Writing – review & editing. **Meimei Han:** Investigation, Writing – review & editing. **Yang Gu:** Investigation, Writing – review & editing. **Qing Shang:** Methodology, Resources, Supervision, Writing – review & editing. **Qinghe Xing:** Conceptualization, Funding acquisition, Methodology, Supervision, Writing – review & editing, Writing – original draft.

## Conflict of interests

The authors declare that they have no known competing financial interests or personal relationships that could have appeared to influence the work reported in this paper.
