# Supplementary material for: Clinically relevant variants detected in Chinese children with global developmental delay/intellectual disability: An exome-wide sequencing study
Source: Genes Dis. 2024 Aug 9;12(4):101389. doi: 10.1016/j.gendis.2024.101389 (PMC11982971; doi:10.1016/j.gendis.2024.101389)
Supplement: Multimedia component 1 [file mmc1.docx]

**Methods and Materials**

**Table S1. The clinical characteristics of 211 children with GDD/ID**

| Clinical Characteristics | Exposed | | Data Unavailable | |
| --- | --- | --- | --- | --- |
|  | Number | % | Number | % |
| GDD (＜60 months) | 179 | 84.8% | 0 | 0.0% |
| ID (≥60 months) | 32 | 15.2% | 0 | 0.0% |
| Male | 137 | 64.9% | 0 | 0.0% |
| Female | 74 | 35.1% | 0 | 0.0% |
| Advanced maternal age (≥35 years old) | 23 | 10.9% | 0 | 0.0% |
| History of miscarriage | 52 | 24.6% | 22 | 10.4% |
| Threatened preterm labor | 26 | 12.3% | 2 | 0.9% |
| Gestational hypertension | 10 | 4.7% | 2 | 0.9% |
| Gestational diabetes | 8 | 3.8% | 0 | 0.0% |
| Prenatal anemia | 37 | 17.5% | 3 | 1.4% |
| Infection during pregnancy | 18 | 8.5% | 4 | 1.9% |
| Intrauterine growth retardation | 11 | 5.2% | 0 | 0.0% |
| Umbilical cord around neck | 35 | 16.6% | 4 | 1.9% |
| Amniotic fluid contamination | 16 | 7.6% | 4 | 1.9% |
| Low birth weight (<2500g) | 15 | 7.1% | 4 | 1.9% |
| Fetal macrosomia(≥4000g) | 17 | 8.1% | 4 | 1.9% |
| Premature birth (<37w) | 17 | 8.1% | 0 | 0.0% |
| Cesarean section | 113 | 53.6% | 1 | 0.5% |
| Neonatal encephalopathy | 14 | 6.6% | 2 | 0.9% |
| Neonatal respiratory distress | 24 | 11.4% | 2 | 0.9% |
| Neonatal sepsis | 4 | 1.9% | 1 | 0.5% |
| Neonatal intracranial hemorrhage | 6 | 2.8% | 0 | 0.0% |
| Family history - First rank relatives | 19 | 9.0% | 1 | 0.5% |
| Abnormal MRI | 123 | 58.3% | 14 | 6.6% |

**Table S3. The exome CNVs related to GDD/ID**

| Sample | Sex | Month Age | CNV | Length | Pathogenic Genes | ACMG Classification |
| --- | --- | --- | --- | --- | --- | --- |
| 2515 | M | 11 | chr15:22738180-28957469del | 6.22 Mb | *SNRPN, NDN* | P |
| 2524 | F | 26 | chr15:23605426-28544662del | 4.94 Mb | *SNRPN, NDN* | P |
| 2581 | M | 32 | chr15:23609489-28566579dup | 4.96 Mb | *SNRPN，NDN* | P |
| 2688 | F | 43 | chr15:22736336-30697401del | 7.96 Mb | *SNRPN, NDN* | P |
| 2557 | M | 36 | chr22:47393529-51220722del | 3.83 Mb | *SHANK3* | P |
| 2582 | M | 17 | chr22:45197956-51216409del | 6.02 Mb | *SHANK3* | P |
| 2598 | M | 3 | chr22:18893887-21576235del | 2.68 Mb | *TBX1* | P |
| 2553 | F | 32 | chrX:140270765-148627429del | 8.36 Mb | *AFF2, FMR1,IDS* | P |
| 2590 | M | 8 | chrX:153626860-153736928dup | 110.07 Kb | *GDI1* | P |
| 2684 | M | 13 | chrX:102884844-103295431dup | 410.59 Kb | *PLP1* | P |
| 2626 | M | 27 | chr2:239036262-242815426del | 3.78 Mb | *TWIST2, KIF1A* | LP |
| 2701 | F | 55 | chr2:200136933-201535402del | 1.40 Mb | *SATB2* | P |
| 2523 | M | 36 | chr12:20522218-23757467del | 3.24 Mb | *SOX5* | LP |
| 2527 | M | 16 | chr12:99222951-111772514del | 12.55 Mb | *CUX2* | P |
| 2704 | M | 11 | chr1:242383289-245018346del | 2.64 Mb | *AKT3, ZNF238* | P |
| 2696 | F | 73 | chr4:68338322-78873761del | 10.54 Mb | *ANKRD17* | LP |
| 2531 | F | 23 | chr11:62847411-64607043del | 1.76 Mb | *KCNK4* | LP |
| 2651 | M | 28 | chr16:88851308-89288591dup | 437.28 Kb | *CDH15* | LP |
| 2518 | M | 7 | chr21:1-48129895dup | 48.13 Mb | *SON, DYRK1A, SIK1* | P |

**Table S4. The available clinical management for 7 diagnosed children with GDD/ID**

| Sample | Sex | Month Age | Genetic Diagnosis | Clinical Phenotype | Clinical Management |
| --- | --- | --- | --- | --- | --- |
| 2491 | M | 14 | ***GALC***:  c.1901T>C, p.Leu634Ser | GDD, thin corpus callosum | Transplantation of allogeneic hematopoietic stem cell or unrelated donors umbilical cord blood |
| 2668 | M | 18 | ***GAMT***:  c.289delC, p.Gln97fs*17 | GDD, Abnormal blood C3, C3/Met and urine 3-hydroxyisovalerate, abnormal auditory pathway conduction, increased auditory threshold | Creatine-monohydrate, L-ornithine, sodium benzoate, and protein/arginine-restricted diets. |
| 2670 | F | 22 | ***TREX1***: c.-26-1(IVS1)G>A;  c.388G>A, p.Asp130Asn; | GDD, abnormal white matter signals in both cerebral hemispheres | Oral baricitinib |
| 2516 | M | 19 | ***ERCC8***:c.320_321insAGTG,  p.Trp107fs*1 | GDD | carbidopa-levodopa |
| 2517 | M | 50 | ***SCN2A***:  c.2548C>T, p.Arg850* | GDD, wider bilateral top subarachnoid space and cerebral sulci | 5-hydroxytryptophan, levodopa, carbidopa, and dopa agonist |
| 2547 | F | 38 | ***SCN2A***:  c.4205A>C, p.Lys1402Thr | GDD, autism | 5-hydroxytryptophan, levodopa, carbidopa, and dopa agonist |
| 2633 | M | 78 | ***SCN2A***:  c.5059A>G, p.Arg1687Gly | ID | 5-hydroxytryptophan, levodopa, carbidopa, and dopa agonist |

**Table S5.** The diagnosis rates of GDD/ID children with different risk factors

| Risk factors | Exposed | | Unexposed | | P Value |
| --- | --- | --- | --- | --- | --- |
|  | Total Number | Diagnosed Number (%) | Total Number | Diagnosed Number (%) |  |
| Neonatal intracranial hemorrhage | 6 | 0(0.0%) | 205 | 83(40.5%) | 0.083 |
| Neonatal encephalopathy | 14 | 2(14.3%) | 195 | 81(41.5%) | **0.044** |
| Infection during pregnancy | 18 | 4(22.2%) | 189 | 79(41.8%) | 0.105 |
| Premature birth (<37 weeks) | 17 | 4(23.5%) | 194 | 79(40.7%) | 0.164 |
| Neonatal respiratory distress | 24 | 7(29.2%) | 185 | 75(40.5%) | 0.283 |
| Gestational hypertension | 10 | 3(30.0%) | 199 | 80(40.2%) | 0.755 |
| Advanced maternal age  (≥35 years old) | 23 | 7(30.4%) | 188 | 76(40.4%) | 0.355 |
| Umbilical cord around neck | 35 | 11(31.4%) | 172 | 70(40.7%) | 0.306 |
| Low birth weight (<2500g) | 15 | 5(33.3%) | 192 | 76(39.6%) | 0.633 |
| Amniotic fluid contamination | 16 | 6(37.5%) | 191 | 75(39.3%) | 0.889 |
| Threatened preterm labor | 26 | 11(42.3%) | 183 | 71(38.8%) | 0.732 |
| Prenatal anemia | 37 | 16(43.2%) | 171 | 67(39.2%) | 0.647 |
| Fetal macrosomia(≥4000g) | 17 | 8(47.1%) | 190 | 73(38.4%) | 0.484 |
| Abnormal MRI | 123 | 59(48.0%) | 74 | 20(27.0%) | **0.004** |
| Gestational diabetes | 8 | 4(50.0%) | 203 | 79(38.9%) | 0.715 |
| Intrauterine growth retardation | 11 | 7(63.6%) | 200 | 76(38.0%) | 0.168 |

**Table S6.** Twenty-one LOF variants across 20 genes

| Sample ID | Sex | Age in Month | Genes | PLI Value | Oe (90% CI) | Brain Mean RPKM | Variations |
| --- | --- | --- | --- | --- | --- | --- | --- |
| 2602 | M | 60 | AGAP2 | 0.9982 | 0.14 (0.08 - 0.27) | 36.162 ± 21.556 | NM_001122772:c.698dupC:p.V234Gfs*41 |
| 2644 | M | 22 | SMARCA1 | 1.0000 | 0.04 (0.02 - 0.14) | 12.838 ± 0.569 | NM_001282874:c.2082_2083del:p.E697Kfs*8 |
| 2655 | M | 7 | SSH2 | 0.9968 | 0.16 (0.1 - 0.28) | 2.324 ± 0.278 | NM_001282129:c.3932delC:p.P1311Lfs*25 |
| 2644 | M | 22 | SSH2 | 0.9968 | 0.16 (0.1 - 0.28) | 2.324 ± 0.278 | NM_001282129:c.745 G>T:p.E249* |
| 2507 | M | 17 | DLGAP3 | 1.0000 | 0.03 (0.01 - 0.14) | 6.468 ± 2.455 | NM_001080418:c.427 C>T:p.R143* |
| 2539 | M | 36 | SCAI | 0.9994 | 0.12 (0.06 - 0.24) | 4.567 ± 2.156 | NM_001144877:c.643 C>T:p.R215* |
| 2652 | M | 4 | ARHGAP30 | 0.9999 | 0.09 (0.04 - 0.21) | 2.368 ± 0.619 | NM_001025598:c.1948delG:p.E650Kfs*20 |
| 2565 | F | 15 | CDC42BPA | 0.9931 | 0.19 (0.13 - 0.28) | 8.647 ± 0.969 | NM_003607:c.4489delT:p.Y1497Ifs*13 |
| 2648 | F | 15 | DENND1A | 0.9998 | 0.11 (0.06 - 0.23) | 3.507 ± 0.434 | NM_001352964:c.868-1->CCA |
| 2546 | M | 5 | FBXO21 | 0.9754 | 0.15 (0.08 - 0.31) | 21.379 ± 1.458 | NM_015002:c.1542 C>A:p.Y514* |
| 2562 | F | 29 | GLG1 | 1.0000 | 0.12 (0.07 - 0.21) | 18.851 ± 2.593 | NM_001145667:c.1408 C>T:p.R470* |
| 2504 | F | 11 | GRM4 | 0.9931 | 0.14 (0.07 - 0.28) | 0.484 ± 0.187 | NM_001256813c.51 G>A:p.W17* |
| 2620 | M | 5 | HERPUD2 | 0.9470 | 0.13 (0.06 - 0.34) | 6.116 ± 1.029 | NM_022373:c.494+1G>T |
| 2692 | F | 84 | HIVEP3 | 0.9060 | 0.2 (0.14 - 0.31) | 1.087 ± 0.417 | NM_024503:c.4260delG:p.S1421Vfs*30 |
| 2573 | F | 52 | LRCH2 | 0.9971 | 0.08 (0.03 - 0.24) | 2.85 ± 0.635 | NM_020871:c.1140_1143del:p.R380Sfs*10 |
| 2503 | M | 80 | SNRNP70 | 0.9993 | 0.04 (0.01 - 0.19) | 30.391 ± 3.102 | NM_003089:c.657 C>G:p.Y219* |
| 2661 | F | 6 | TAF1L | 0.9618 | 0.18 (0.11 - 0.32) | / | NM_153809:c.54delC:p.I19Sfs*19 |
| 2675 | F | 156 | TOX3 | 0.9995 | 0.04 (0.01 - 0.18) | 1.866 ± 1.176 | NM_001080430:c.1505delA:p.N502Ifs*39 |
| 2563 | M | 36 | TSHZ2 | 0.9814 | 0.15 (0.07 - 0.31) | 0.873 ± 0.478 | NM_173485:c.1080 T>G:p.Y360* |
| 2618 | F | 41 | USP48 | 1.0000 | 0.08 (0.04 - 0.16) | 5.458 ± 0.352 | NM_032236:c.2623-1->A |
| 2641 | M | 4 | ZNF608 | 1.0000 | 0.06 (0.03 - 0.17) | 4.127 ± 2.358 | NM_020747:c.4050_4051insCT:p.Y1351Lfs*20 |

**Methods**

***GDD/ID Children Recruitment and Clinical Information Collecting***

A total of 211 children with GDD/ID from Henan Children's Hospital was recruited for this study, comprised of 166 trios, 35 duos with one parent, and 10 children alone. Blood samples were collected from the enrolled children and their parents. Informed consent was obtained from the patients’ legal guardians after a full explanation of the procedure was provided. The research protocol was reviewed and approved by the Ethics Committee of the Children’s Hospital of Fudan University (2019-070) and was performed in accordance with the Declaration of Helsinki.

Clinical information was obtained from questionnaires completed by the participants or by their guardians, a clinical review was also conducted by a treating clinician to determine demographic variables (sex, age, and gestational age) and GDD/ID risk factors (such as maternal age, pregnancy-induced hypertension, infection during pregnancy, birth asphyxia, birth weight, and neonatal encephalopathy) (Supplementary Table S1).

***WES and Data Analysis***

Genomic DNA was extracted from the peripheral blood using a QIAamp® DNA Blood Mini Kit (QIAGEN, Hilden, Germany) according to the manufacturer’s protocol. DNA libraries were prepared using xGen Exome Research Panel v1.0 (Integrated DNA Technologies, Coralville, IA, USA). Sequencing was performed on an Illumina NovaSeq 6000 system (San Diego, CA, USA) with an average coverage of 100X. Paired-end sequencing reads were mapped to the hg19 human reference genome using BWA, and variants were identified using GATK. SAMtools and Pindel were used to call SNVs and insertions/deletions, respectively. Variants with a minor allele frequency of >1% in the dbSNP, ExAC database, NHLBI exome sequencing project, 1,000 Genomes Project database, and 1000 in-house controls were removed. The pathogenicity of the variants was predicted using SIFT, PolyPhen-2, and CADD. Candidate disease-causing variants were confirmed using Sanger sequencing. To determine whether the origin of the variants was paternal, maternal, or *de novo*, candidate variants were genotyped from the parents’ DNA. Pathogenic (P) and likely pathogenic (LP) variants identified in known neurodevelopmental disease genes recorded in OMIM were considered as disease-causing variants according to American College of Medical Genetics guidelines, appropriate inheritance patterns, and disease-phenotype concordance ^1^.

***CNV analysis***

eXome-Hidden Markov model software and principal component analysis were used to remove sequencing noise ^2,3^. The CNV Kit fix module was used for GC and bias correction. CANOES and ExomeDepth were separately applied to detect CNVs in the WES data, and the results were merged. CNVs were clinically interpreted according to American College of Medical Genetics guidelines.

***Statistics***

Statistical analyses were performed using R software v.3.5.4 (R Foundation for Statistical Computing, Vienna, Austria). Statistical significance was defined as P < 0.05. All tests were 2-sided. The presence or absence of various risk factors was coded as binary variables. The associations of risk factors with the genetic diagnostic rate were assessed by using Pearson χ² tests and Fisher exact test (SPSS software, version 18.0, SPSS, Inc., Chicago, IL, USA).

**References**

1. Qiao Y, Gu Y, Cheng Y, et al. Case Report: Novel MFSD8 Variants in a Chinese Family With Neuronal Ceroid Lipofuscinoses 7. *Front Genet.* 2022;13:807515.

2. Fromer M, Moran JL, Chambert K, et al. Discovery and statistical genotyping of copy-number variation from whole-exome sequencing depth. *Am J Hum Genet.* 2012;91(4):597-607.

3. Xiang J, Ding Y, Yang F, et al. Genetic Analysis of Children With Unexplained Developmental Delay and/or Intellectual Disability by Whole-Exome Sequencing. *Front Genet.* 2021;12:738561.
